# Transcriptomic analysis of rice in response to iron deficiency and excess

**DOI:** 10.1186/s12284-014-0018-1

**Published:** 2014-09-12

**Authors:** Khurram Bashir, Kousuke Hanada, Minami Shimizu, Motoaki Seki, Hiromi Nakanishi, Naoko K Nishizawa

**Affiliations:** Laboratory of Plant Biotechnology, Department of Global Agricultural Sciences, Graduate School of Agricultural and Life Sciences, The University of Tokyo, 1-1-1 Yayoi, Bunkyo-ku, Tokyo, 113-8657 Japan; Plant Genomics Network Research Team, Center for Sustainable Resource Science, RIKEN Yokohama Campus, 1-7-22 Suehiro-cho, Tsurumi-ku, Yokohama City, 230-0045 Kanagawa, Japan; Gene Discovery Research Group, Center for Sustainable Resource Science, RIKEN Yokohama Campus, 1-7-22 Suehiro-cho, Tsurumi-ku, Yokohama City, 230-0045 Kanagawa, Japan; Frontier Research Academy for Young Researchers, Department of Bioscience and Bioinformatics, Kyusyu Institute of Technology, Iizuka, 820-8502 Fukuoka, Japan; Kihara Institute for Biological Research, Yokohama City University, 22-2 Seto, Kanazawa-ku, Yokohama, 236-0027 Japan; Research Institute for Bioresources and Biotechnology, Ishikawa Prefectural University, 1-308 Suematsu, Nonoichi-shi, 921-8836 Ishikawa, Japan

**Keywords:** Excess Fe, Fe deficiency, Iron, Peptides, Rice, Small open reading frames

## Abstract

Iron (Fe) is essential micronutrient for plants and its deficiency as well as toxicity is a serious agricultural problem. The mechanisms of Fe deficiency are reasonably understood, however our knowledge about plants response to excess Fe is limited. Moreover, the regulation of small open reading frames (sORFs) in response to abiotic stress has not been reported in rice. Understanding the regulation of rice transcriptome in response to Fe deficiency and excess could provide bases for developing strategies to breed plants tolerant to Fe deficiency as well as excess Fe.

**Results:**

We used a novel rice 110 K microarray harbouring ~48,620 sORFs to understand the transcriptomic changes that occur in response to Fe deficiency and excess. In roots, 36 genes were upregulated by excess Fe, of which three were sORFs. In contrast, 1509 genes were upregulated by Fe deficiency, of which 90 (6%) were sORFs. Co-expression analysis revealed that the expression of some sORFs was positively correlated with the genes upregulated by Fe deficiency. In shoots, 50 (19%) of the genes upregulated by Fe deficiency and 1076 out of 2480 (43%) genes upregulated by excess Fe were sORFs. These results suggest that excess Fe may significantly alter metabolism, particularly in shoots.

**Conclusion:**

These data not only reveal the genes regulated by excess Fe, but also suggest that sORFs might play an important role in the response of plants to Fe deficiency and excess.

**Electronic supplementary material:**

The online version of this article (doi:10.1186/s12284-014-0018-1) contains supplementary material, which is available to authorized users.

## Background

Iron (Fe) is an essential micronutrient for all higher organisms, and its deficiency causes a serious nutritional problem in both humans and plants. Although mineral soils are rich in Fe (>5%), various factors such as a high soil pH and the presence of sodium carbonate adversely affect the availability and uptake of Fe through plant roots (Marschner [1995]; Mori [1999]). In contrast, a low soil pH and anaerobic conditions, such as in a paddy field, lead to the reduction of Fe^3+^ to Fe^2+^, which can result in increased absorption and conditions of excess Fe (Neue et al. [1998]; Quinet et al. [2012]). Fe toxicity can occur in flooded soils with a pH below 5.8 under aerobic conditions, and at a pH below 6.5 under anaerobic conditions (Fageria et al. [2008]). Fe toxicity is a serious agricultural problem, particularly when plants are grown in acidic soils (Guerinot and Ying [1994]; Quinet et al. [2012]). Developing plants that can grow in problematic soils requires an understanding of the molecular mechanisms of Fe uptake, transport, and storage in plants under conditions of varying Fe availability (Bashir et al. [2013a]). The molecular mechanisms of Fe uptake from soil have been extensively studied (Bashir et al. [2010]; Bashir et al. [2011b]; Bashir et al. [2013a]; Guerinot [2010]; Guerinot and Ying [1994]; Ishimaru et al. [2011b]; Ishimaru et al. [2011a]; Kobayashi and Nishizawa [2012]; Marschner [1995]). Plants are divided into two broad categories (strategies I and II) based on how they uptake Fe from the soil (Marschner [1995]; Marschner and Römheld [1994]). Rice is a strategy II plant, and secretes 2'-deoxymugineic acid (DMA) to acquire soil Fe. The genes involved in DMA synthesis have been cloned and characterized (Bashir et al. [2006]; Bashir and Nishizawa [2006]; Inoue et al. [2003]; Inoue et al. [2008]; Nozoye et al. [2004]; Suzuki et al. [2006]; Suzuki et al. [2008]; Suzuki et al. [2012]; Takahashi et al. [1999]). Specifically, L-methionine is converted to nicotianamine (NA) by NA synthase 1-3 (OsNAS1-3), and is then converted to 3'-keto acid by NA aminotransferase 1 (OsNAAT1) and finally DMA synthase (OsDMAS1) converts this 3'-keto acid to DMA (Bashir et al. [2006]; Bashir and Nishizawa [2006]; Bashir et al. [2010]; Inoue et al. [2003]; Inoue et al. [2008]; Ma et al. [1995]; Ma et al. [1999]; Mori and Nishizawa [1987]; Nozoye et al. [2014a]; Nozoye et al. [2014b]). DMA is then secreted to the rhizosphere via the mugineic acid transporter (OsTOM1) Nozoye et al. [2011]. In the rhizosphere, DMA binds to Fe(III), and the resulting DMA-Fe (III) complex is taken up by OsYSL15 (Inoue et al. [2009]; Lee et al. [2009]). Rice also uses OsIRT1 to uptake ferrous Fe under paddy field conditions, and secretes phenolics to solubilize apoplasmic Fe (Bashir et al. [2011b]; Ishimaru et al. [2011a]; Ishimaru et al. [2011b]). Once Fe is absorbed through roots, it is translocated to the aerial parts of the plant. The genes involved in root-to-shoot translocation and the transport of Fe to subcellular organelles have also been characterized (Aoyama et al. [2009]; Bashir et al. [2011a]; Bashir et al. [2011c]; Bashir et al. [2013b]; Ishimaru et al. [2009]; Ishimaru et al. [2010]; Ishimaru et al. [2011a]; Ishimaru et al. [2011b]; Ishimaru et al. [2012]; Kakei et al. [2012]; Koike et al. [2004]; Lee et al. [2012]; Yokosho et al. [2009]; Zhang et al. [2012b]).

Plants can accumulate varying levels of Fe and the response of rice to Fe toxicity was recently summarized after comprehensive transcriptomic and physiological analyses (Quinet et al. [2012]). In the current study, our main objective was to understand the transcriptomic response of rice to different conditions of Fe availability. We therefore performed a microarray analysis of plants accumulating high, yet not physiologically toxic, levels of Fe. Although the rice genome has been sequenced (Kawahara et al. [2013]), the identification of small open reading frames (sORFs) typically consisting of fewer than 100 codons was not addressed in plants until recently (Hanada et al. [2013]; Hanada et al. [2010]; Hanada et al. [2007]). These sORFs play a critical role in morphogenesis in *Arabidopsis thaliana* (Hanada et al. [2013]; Hanada et al. [2010]; Hanada et al. [2007]). Although the potential role of sORF in rice is recently discussed (Okamoto et al. [2014]) their regulation in response to different abiotic stresses has not been assessed in rice. In this study, we used a novel 110 K rice microarray that, along with previously identified genes, includes ~48,620 sORFs to identify transcriptional changes in response to Fe deficiency and excess in rice roots and shoots. This will allow a better understanding of the response of plants to these stresses, and suggests the involvement of sORFs in Fe metabolism under different conditions of Fe availability.

## Results

### Morphological responses to Fe deficiency and excess Fe

**Figure 1:**
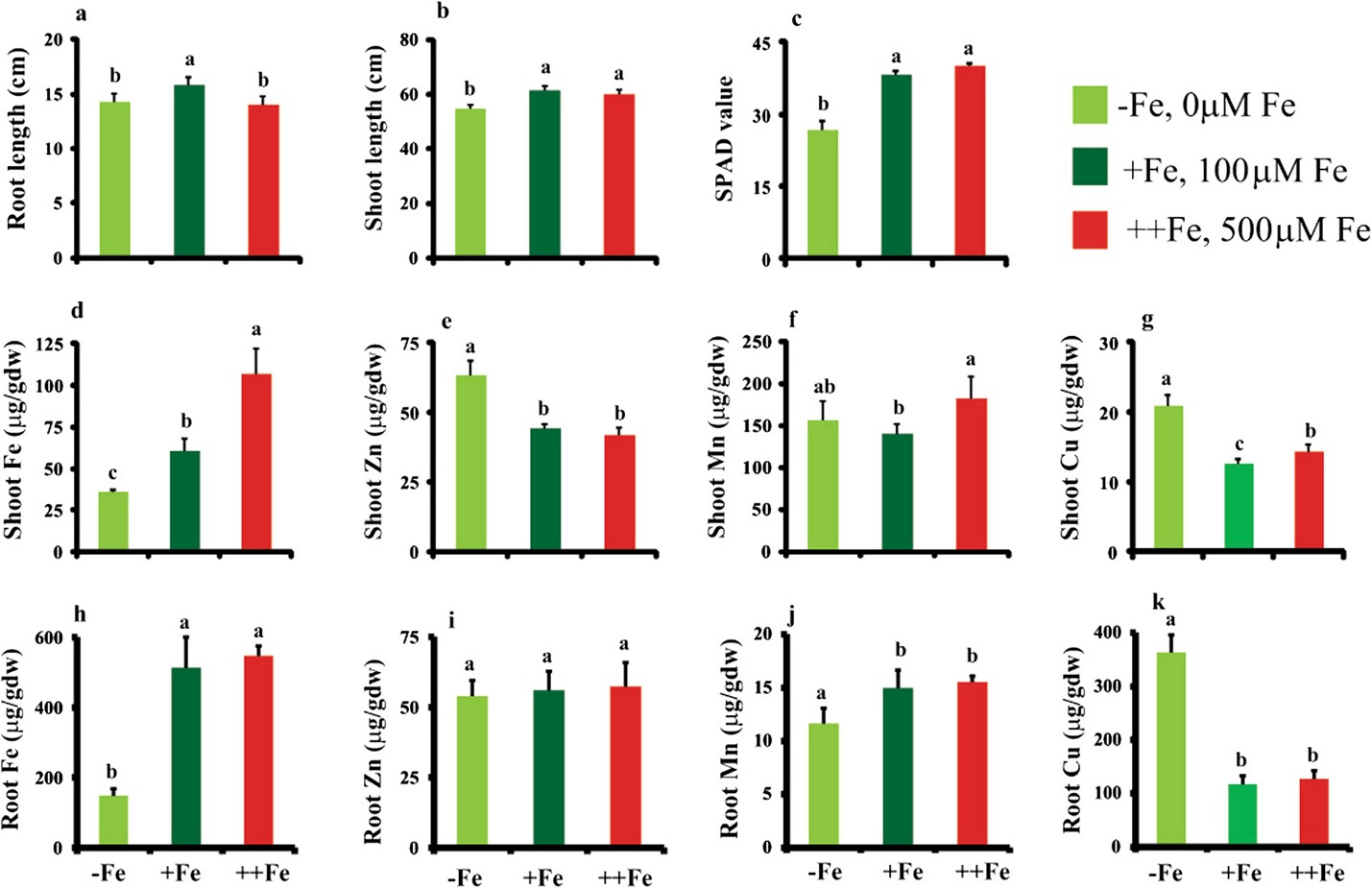
**Morphological characteristics and metal profiling of plants grown under conditions of Fe deficiency and excess. a)** Root length (cm). **b)** Shoot length (cm). **c)** Chlorophyll content. **d)** Shoot Fe. **e)** Shoot Zn. **f)** Shoot Mn. **g)** Shoot Cu. **h)** Root Fe. **i)** Root Zn. **j)** Root Mn. **k)** Root Cu (μg/g dry weight). Vertical bars followed by different letters are significantly different from each other, according to the Tukey-Kramer test (*p* < 0.05; *n* = 4).

When plants were grown under Fe-deficient conditions, the root and shoot length as well as the chlorophyll content decreased significantly compared with plants grown in the presence of 100 μM Fe-EDTA (Figure 1a-c). In contrast, when plants were grown under conditions of excess Fe, the root length was reduced, but no significant differences were observed in plant height or chlorophyll content compared with wild-type plants (Figure 1a-c). In the shoots of Fe-deficient plants, the concentrations of Fe were 50% lower than in plants grown with 100 μM Fe, whereas plants grown under conditions of excess Fe accumulated two-fold more Fe in their leaves (Figure 1d).

In plants grown under Fe-deficient conditions, the concentrations of zinc and copper (Cu) increased in the shoots, whereas the manganese (Mn) concentrations were comparable to plants grown in the presence of Fe (Figure 1e-g). In contrast, plants grown in the presence of excess Fe accumulated more Mn in their shoots compared to plants supplied with 100 μM Fe (Figure 1f). In the roots of plants grown under Fe-deficient conditions, the concentrations of Fe and Mn decreased significantly, whereas the concentration of Cu increased compared to plants grown with 100 μM Fe (Figure 1h-k).

### Genes upregulated by Fe deficiency and downregulated by excess Fe in roots

**Figure 2:**
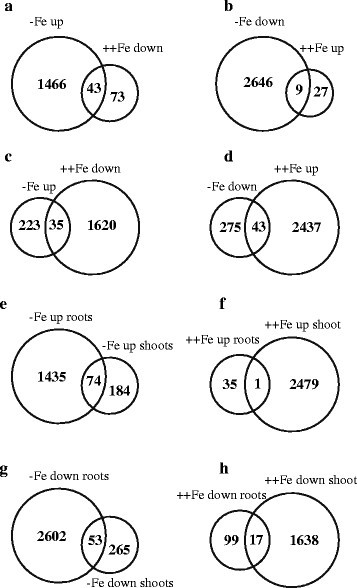
**Venn diagram representing the transcriptional changes in response to Fe deficiency and excess. a)** Number of genes upregulated by Fe deficiency and downregulated by excess Fe in roots. **b)** Number of genes downregulated by Fe deficiency and upregulated by excess Fe in roots. **c)** Number of genes upregulated by Fe deficiency and downregulated by excess Fe in shoots. **d)** Number of genes downregulated by Fe deficiency and upregulated by excess Fe in shoots. **e)** Genes upregulated by Fe deficiency both in roots and shoots. **f)** Genes upregulated by excess Fe both in roots and shoots. **g)** Genes downregulated by Fe deficiency both in roots and shoots. **h)** Genes downregulated by excess Fe both in roots and shoots.

**Figure 3:**
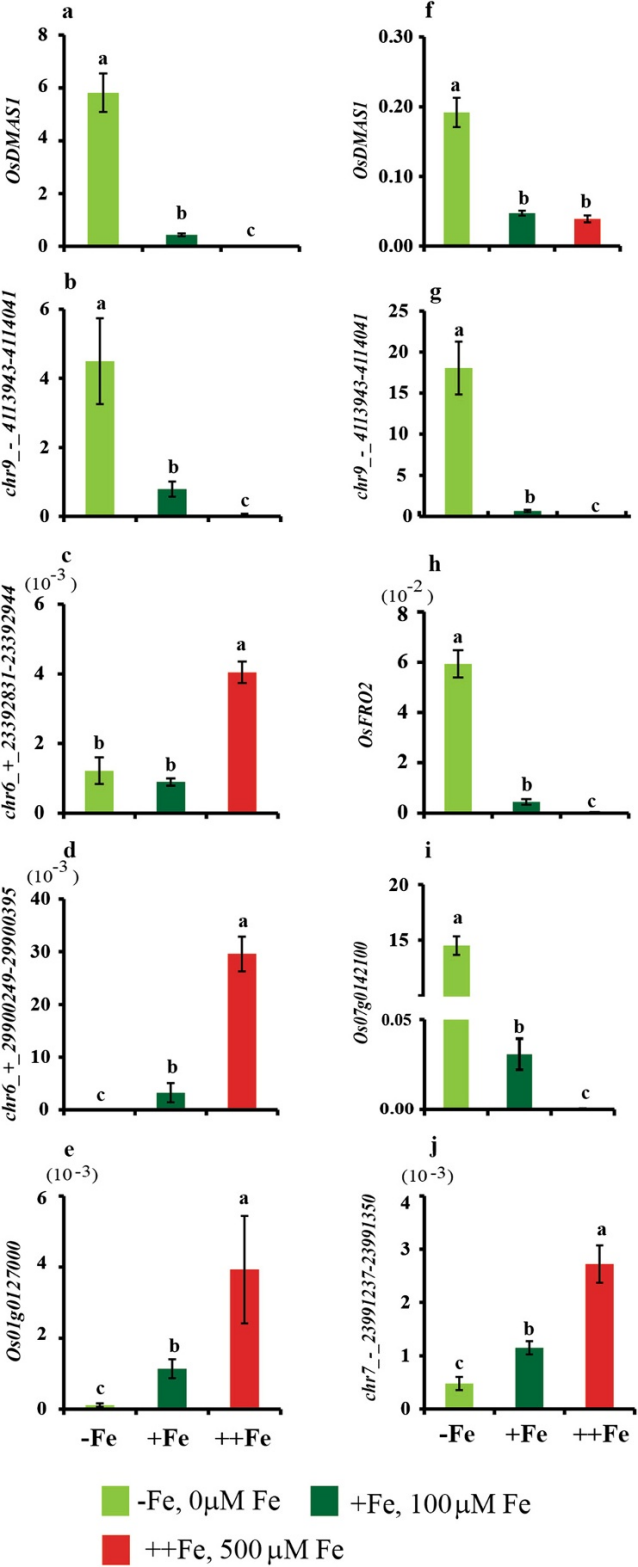
**Expressionanalysis of selected genes in response to varying Fe availability.** Expression of **a, f)** *OsDMAS1*. **b, g)** *chr9_-_4113943-4114041*. **c)** *chr6_ + _23392831-23392944*. **d)** *chr6_ + _29900249-29900395*. **e)** *Os01g0127000*. **h)** *OsFRO2*. **i)** *Os07g0142100*. **j)** *chr7_-_23991237-23991350*. **a-e)** Root. **f-g)** Shoot. The graph shows mean ± s.d. relative to the expression of α-tubulin. Vertical bars followed by different letters are significantly different from each other, according to the Tukey-Kramer test (*p* < 0.05; *n* = 3).

Several studies reported the upregulation of genes in response to Fe deficiency in rice (Bashir et al. [2013c]; Bashir and Nishizawa [2013]; Ishimaru et al. [2009]; Nozoye et al. [2011]), however little attention is paid to identify genes regulated by excess Fe. Before carrying out our microarray analysis, we used RT-PCR to assess the expression of *OsDMAS1* and *OsVIT2* to confirm the effects of excess Fe and deficiency treatments. *OsDMAS1* is upregulated by Fe deficiency, while expression of vacuolar Fe transporter *OsVIT2* is reported to be upregulated by excess Fe (Bashir et al. [2011c]; Zhang et al. [2012b]; Bashir et al. [2013b]). In our study, *OsDMAS1* was upregulated by Fe deficiency in both roots and shoots, and was downregulated by excess Fe. As expected, the expression of *OsVIT2* was upregulated by excess Fe in both shoots and roots (Additional file 1: Figure S1). In general, the transcriptomic changes in roots were clearer in response to Fe-deficiency as compared to excess Fe. On the other hand in shoot tissue, the expression of secondary metabolism related genes was more significantly altered by excess Fe compared to Fe deficiency. Our microarray results revealed the upregulation of 1509 genes in response to Fe deficiency in roots (Figure 2a, e and Additional file 2: Table S1), of which 90 (6%) were sORFs. In addition, 116 genes were downregulated by excess Fe (Figure 2a, Additional file 2: Table S2). Of the 1509 genes upregulated by Fe deficiency, 43 were downregulated by excess Fe (Figure 2a, Table 1). The genes presented in Table 1 are therefore highly responsive to Fe availability in roots. Consistent with previous microarray reports, the genes upregulated by Fe deficiency included those involved in the synthesis of DMA such as *OsNAAT1*, and *OsDMAS1*, those involved in Fe-NA or DMA complex transport (*OsYSL2* and *OsYSL15*), and the DMA efflux transporter (*OsTOM1*) (Table 1 (Ishimaru et al. [2009])). In addition, *OsIRO2* and two other basic helix loop helix (bHLH)-type transcription factors were upregulated by Fe deficiency (Tables 1 and S1). Two ABC transporters that are upregulated by excess Cu (Lin et al. [2013]), were upregulated by Fe deficiency as were two amino acid transporters, of which *Os02g0788800* is also upregulated by excess Cu (Lin et al. [2013]). MapMan analysis revealed that many metabolic genes were upregulated or downregulated in response to Fe deficiency, and many of these were upregulated in response to excess Fe in roots (Additional file 1: Figure S2**)**. Changes in the expression of *OsDMAS1* and sORF *chr9_-_4113943-4114041* were also confirmed through real time PCR and the data was in line with microarray analysis (Figure 3).

**Table 1:** **Genes upregulated by Fe-deficiency and downregulated by excess Fe in roots**

Locus	Gene	-Fe/+Fe	-Fe/+Fe	++Fe/+Fe	++Fe/+Fe
Os02g0306401	OsNAAT1	4.072	4.665	0.211	0.160
Os03g0237100	OsDMAS1	6.852	3.768	0.109	0.096
Os02g0649900	OsYSL2	53.191	54.448	0.439	0.498
Os02g0650300	OsYSL15	5.035	6.444	0.125	0.094
Os11g0134900	OsTOM1	10.903	5.724	0.078	0.064
Os03g0667300	OsIRT2 **(0.67)**	8.568	10.120	0.183	0.107
Os01g0952800	OsIRO2 **(0.89)** ***(0.91)***	2.394	3.313	0.038	0.029
Os12g0282000	MIR **(0.848),** ***(0.94)***	13.538	10.484	0.100	0.084
Os12g0570700	OsIDS1	5.992	9.164	0.370	0.318
Os03g0751100	OPT **(0.91)** ***(0.82)***	3.410	2.244	0.147	0.091
Os01g0871600	TGF-beta receptor, type I/II **(0.74)** ***(0.79)***	12.263	8.808	0.045	0.036
Os10g0567400	Rieske_[2Fe-2S]_region_domain_containing_protein	7.620	5.157	0.370	0.325
Os08g0527700	TGF-beta_receptor,_type_I/II_extracellular_region_family_protein **(0.80)** ***(0.83)***	5.608	6.219	0.391	0.241
Os01g0871500	TGF-beta_receptor,_type_I/II_extracellular_region_family_protein **(0.86)** ***(0.89)***	3.336	2.311	0.246	0.198
Os09g0129600	Site-specific_recombinase_family_protein	7.529	6.035	0.275	0.197
Os04g0306400	Ribose_5-phosphate_isomerase_family_protein	2.639	2.213	0.276	0.202
Os03g0439700	Protein_of_unknown_function_DUF1230_family_protein **(0.81),** ***(0.89)***	7.024	8.104	0.071	0.055
Os01g0655500	Protein_kinase-like_domain_containing_protein **(0.80)** ***(0.88)***	5.218	3.746	0.157	0.106
Os01g0494300	Non-protein_coding_transcript,_putative_npRNA **(0.72)** ***(0.74)***	4.468	5.140	0.470	0.400
Os12g0236200	Non-protein_coding_transcript,_unclassifiable_transcript **(0.82)**	30.420	11.722	0.058	0.132
Os02g0707633	NONE Category **(0.81)** ***(0.85)***	11.858	8.096	0.051	0.042
Os09g0118650	NONE Category **(0.98)** ***(0.88)***	7.410	7.636	0.048	0.043
Os02g0779400	NONE Category	6.789	3.371	0.156	0.131
Os12g0508500	NONE Category	6.502	7.113	0.142	0.135
Os03g0615600	NONE Category **(0.91)** ***(0.96)***	4.090	3.454	0.061	0.049
Os12g0435466	NONE Category **(0.81)** ***(0.85)***	11.679	5.892	0.063	0.052
Os01g0608300	Conserved_hypothetical_protein **(0.98)** ***(0.94)***	11.851	9.647	0.158	0.134
Os11g0262600	Conserved_hypothetical_protein **(0.95)** ***(0.98)***	7.386	6.953	0.044	0.045
Os03g0431600	Conserved_hypothetical_protein **(0.94)** ***(0.99)***	7.084	5.026	0.056	0.042
Os03g0725200	Conserved_hypothetical_protein	7.066	3.573	0.054	0.048
Os10g0195250	Conserved_hypothetical_protein **(0.95)** ***(0.96)***	6.610	5.194	0.040	0.039
Os02g0594600	Conserved_hypothetical_protein	5.792	4.971	0.071	0.065
Os06g0294950	Conserved_hypothetical_protein **(0.96)** ***(0.96)***	5.407	5.059	0.040	0.039
LOC_Os06g19095	Conserved_hypothetical_protein **(0.98)** ***(0.96)***	5.407	5.059	0.040	0.039
Os01g0332200	Conserved_hypothetical_protein	5.279	5.794	0.459	0.203
Os10g0159066	Conserved_hypothetical_protein **(0.80)** ***(0.71)***	4.907	5.267	0.110	0.082
Os05g0554000	Conserved_hypothetical_protein **(0.91)**	4.784	4.416	0.355	0.395
Os01g0689300	Conserved_hypothetical_protein **(0.75)** ***(0.76)***	4.646	4.288	0.511	0.406
Os12g0236100	Conserved_hypothetical_protein **(0.91)** ***(0.95)***	4.560	3.175	0.073	0.061
Os01g0953000	Conserved_hypothetical_protein	3.215	2.956	0.470	0.373
**chr9_-_4113943-4114041**	sORF **(1.00)** ***(0.94)***	6.364	5.478	0.042	0.042
***chr4_-_5708578-5708748***	sORF **(0.94)** ***(1.00)***	5.376	3.090	0.054	0.036
chr1_ + _43772594-43772752	sORF **(0.83)** ***(0.83)***	3.181	4.281	0.249	0.263

The expression of genes listed in Table 1 is up or down regulated at least two fold in both biological replications. Coexpression analysis were done at http://evolver.psc.riken.jp/seiken/OS/co-express.html. This database contains microarray data of 40 different experimental conditions obtained through microarray analysis using the same custom microarray chip as described in this manuscript.

The values written in bold indicate the co-expression coefficient for **chr9_-_4113943-4114041,** while the values written in bold Italic indicate the co-expression coefficient for sORF ***chr4_-_5708578-5708748.***

### Genes upregulated by excess Fe and downregulated by Fe deficiency in roots

In roots, 36 genes were upregulated by excess Fe, of which three were sORFs (Table 2), while 2655 genes were downregulated by Fe deficiency, of which 1225 (46%) were sORFs (Additional file 2: Table S3). However, only nine genes were upregulated by excess Fe and downregulated by Fe deficiency. The genes upregulated by excess Fe included four peroxidases, multi-Cu oxidase (*Os01g0127000*), and alcohol dehydrogenase, suggesting that excess Fe causes oxidative stress. Three cytochrome P450 family proteins, which may play a role in electron transport, were also upregulated, as was the expression of one subtilase family gene. Five uncharacterized proteins and three sORFs genes were also upregulated by excess Fe (Table 2). Changes in the expression of multicopper oxidase *Os01g0127000* and two sORFs chr6_ + _29900249-29900395 and chr6_ + _23392831-23392944 were also confirmed through real time PCR (Figure 3).

**Table 2:** **Genes upregulated by excess Fe in roots**

Locus	Gene	-Fe/+Fe	-Fe/+Fe	++Fe/+Fe	++Fe/+Fe
Os06g0597600	Aromatic-ring_hydroxylase_family_protein	1.695	1.047	2.401	2.054
Os09g0388400	Cof_protein_family_protein	1.528	0.532	4.731	2.708
Os01g0895300	Cytochrome b561, eukaryote domain containing protein	0.369	0.401	2.098	2.163
Os01g0803800	Cytochrome_P450_family_protein	0.397	0.391	5.581	5.668
Os01g0803900	Cytochrome_P450_family_protein	0.760	0.925	6.990	5.304
Os11g0138300	Cytochrome_P450_family_protei	1.590	2.129	6.240	4.700
Os01g0893700	DOMON_related_domain_containing_protein	0.818	0.756	25.037	23.014
Os01g0895200	DOMON_related_domain_containing_protein	0.312	0.205	3.241	2.876
Os06g0695300	Haem_peroxidase,_plant/fungal/bacterial_family_protein	0.148	0.147	9.393	7.456
Os01g0736500	Harpin-induced_1_domain_containing_protein	1.650	1.563	2.312	1.979
Os04g0542000	HAT_dimerisation_domain_containing_protein	1.942	1.176	2.360	2.143
Os04g0469000	Heavy_metal_transport/detoxification_protein	3.068	3.461	1.986	3.308
Os01g0129600	LBD40,	0.539	0.553	2.823	3.143
Os01g0127000	Multicopper_oxidase,copper_ion_binding	0.040	0.038	10.058	10.692
Os07g0681200	Plant_acid_phosphatase_family_protein	0.522	0.414	2.480	2.531
Os05g0253200	Protein_kinase-like_domain_containing_protein	2.880	1.837	2.600	3.229
Os02g0586000	Quinonprotein_alcohol_dehydrogenase-like_domain	1.112	0.772	3.192	3.130
Os01g0941400	Beta-1,3-glucanase	0.790	0.989	1.982	8.542
Os01g0940700	Glucan_endo-1,3-beta-glucosidase	1.219	1.726	2.520	14.386
Os03g0273200	Similar_to_Laccase_(EC_1.10.3.2) copper_ion_binding	5.600	5.213	2.697	3.305
Os03g0234100	Similar to Non-symbiotic hemoglobin 4 (rHb4)	1.286	1.206	2.168	2.152
Os03g0368300	Similar to Peroxidase 1	0.482	0.529	2.593	2.244
Os03g0369000	Similar to Peroxidase 1	0.763	0.738	2.608	2.861
Os07g0531400	Similar to Peroxidase 27 precursor (EC_1.11.1.7)	0.161	0.097	9.404	8.606
Os01g0795100	Similar to Subtilase.";category_	2.699	2.176	7.865	4.144
Os06g0578100	Von Willebrand factor, type A domain containing protein	0.737	1.932	5.243	5.210
Os11g0687100	Von Willebrand_factor, type A domain containing protein	1.657	3.269	4.657	4.324
Os01g0838600	Zinc finger, C2H2-type domain containing proteinc	3.505	3.445	2.704	2.022
Os02g0582900	Conserved hypothetical protein	0.443	0.263	3.946	3.242
Os04g0438600	Conserved hypothetical protein	1.375	1.398	2.466	2.099
Os04g0538300	Conserved_hypothetical_protein	0.147	0.236	18.160	29.066
Os01g0803600	NONE";category_"NONE	0.661	0.783	3.882	3.204
Os10g0451601	NONE";category_"NONE	2.712	2.123	23.277	14.910
chr2_-_1866365-1866487	sORF	5.403	2.693	4.059	2.612
chr6_ + _23392831-23392944	sORF	0.768	1.221	9.650	7.388
chr6_ + _29900249-29900395	sORF	0.106	0.111	7.392	6.886

The expression of genes listed in Table 2 is up regulated at least two fold in both biological replications.

Most of the genes downregulated by Fe deficiency (1225; 46%) were categorized as sORFs. Other downregulated genes include 15 Zn finger proteins, two WRKY transcription factors, 11 peptidase, eight heme peroxidases, and genes involved in the ethylene response and other metabolic pathways such as methionine metabolism (Additional file 2: Table S3).

### Genes upregulated by Fe deficiency and downregulated by excess Fe in shoots

In shoots, 258 genes were upregulated by Fe deficiency, of which 35 genes were also downregulated by excess Fe (Figure 2c). Consistent with previous reports, genes involved in DMA synthesis and transport (such as *OsNAS1-2* and *OsDMAS1*, *OsTOM1*), Fe-NA or DMA complex transport (*OsYSL2*) were upregulated by Fe deficiency (Additional file 2: Table S4). Other genes regulated by Fe deficiency included *OsIRT2*, *OsIDS1*, *OsIRO2* and *OsFRO2.* OsIDS1 is a metallothionein (MT) gene highly responsive to Fe deficiency (Itai et al. [2013]). Of the genes upregulated by Fe deficiency in shoots, 50 (19%) were sORFs, but only two of these were also downregulated by excess Fe, whereas 1655 genes were downregulated by excess Fe (Additional file 2: Table S5). The genes downregulated by excess Fe include NADPH-dependent oxidoreductases and peroxidases. Two bHLH transcription factors, a cyclin-like F-box domain-containing protein, a protein kinase, and two sORFs (*chr6_ + _7967232-7967441* and *chr9_-_4113943-4114041*) were upregulated by Fe deficiency and downregulated by excess Fe (Table 3). A total of 74 genes were upregulated by Fe deficiency in both roots and shoots (Figure 2e), but only 17 genes were downregulated by excess Fe in both roots and shoots (Figure 2h).

**Table 3:** **Genes upregulated by Fe deficiency and downregulated by excess Fe in shoots**

Locus	Gene	-Fe/+Fe	-Fe/+Fe	++Fe/+Fe	++Fe/+Fe
Os03g0379300	bHLH_domain_containing_protein	4.969	7.570	0.108	0.145
Os04g0578600	OsFRO2	5.457	8.123	0.021	0.027
Os01g0655500	Protein_kinase-like_domain_containing_protein	8.077	4.031	0.161	0.077
Os03g0736900	Conserved_hypothetical_protein	2.415	3.145	0.322	0.384
Os07g0438300	Conserved_hypothetical_protein	3.543	3.060	0.428	0.365
Os01g0689300	Conserved_hypothetical_protein	6.633	6.891	0.362	0.325
Os03g0725200	Conserved_hypothetical_protein	7.943	10.426	0.007	0.023
Os10g0159066	Conserved_hypothetical_protein	8.220	13.331	0.081	0.124
Os10g0195250	Conserved_hypothetical_protein	10.161	14.816	0.003	0.024
Os02g0594600	Conserved_hypothetical_protein	11.980	17.788	0.037	0.073
Os06g0294950	Conserved_hypothetical_protein	14.289	17.805	0.002	0.013
LOC_Os06g19095	Conserved_hypothetical_protein	14.289	17.805	0.002	0.013
Os01g0608300	Conserved_hypothetical_protein	16.000	20.626	0.129	0.159
Os11g0262600	Conserved_hypothetical_protein	18.554	29.444	0.026	0.037
Os07g0142100	Conserved_hypothetical_protein	98.700	168.002	0.006	0.044
Os01g0659900	Cyclin-like_F-box_domain_containing_protein	2.137	2.187	0.401	0.476
Os07g0475300	NONE";category_"NONE	2.349	2.029	0.531	0.503
Os02g0746500	NONE";category_"NONE	2.390	2.909	0.425	0.477
Os04g0380900	NONE";category_"NONE	5.670	7.148	0.397	0.464
Os10g0193700	NONE";category_"NONE	7.163	6.096	0.530	0.497
Os09g0118650	NONE";category_"NONE	17.954	27.631	0.004	0.020
Os10g0524300	Peptidoglycan-binding_LysM_domain_containing	3.627	3.952	0.518	0.413
Os05g0592300	Protein_of_unknown_function_DUF1637_family_protein	6.181	6.361	0.159	0.285
Os07g0150100	Protein_of_unknown_function_DUF221_domain	2.185	2.185	0.511	0.435
Os08g0425700	Similar_to_Annexin-like_protein	2.654	1.975	0.431	0.395
Os03g0718800	Similar_to_Physical_impedance_induced_protein	2.118	2.299	0.437	0.288
Os04g0672100	Similar_to_Phytosulfokine_receptor_precursor_(EC_2.7.1.37)	3.066	2.988	0.402	0.452
Os09g0442600	Similar_to_RSH2	6.774	6.480	0.426	0.417
Os05g0566200	Similar_to_Small_CTD_phosphatase_1_splice_variant	2.441	2.265	0.480	0.483
Os01g0871500	TGF-beta_receptor,_type_I/II_extracellular_region_family_protein	2.591	3.208	0.309	0.322
Os01g0871600	TGF-beta_receptor,_type_I/II_extracellular_region_family_protein	36.333	35.382	0.110	0.140
Os09g0442400	t-snare_domain_containing_protein	2.908	2.694	0.311	0.388
Os05g0551000	Zinc_finger,_CHY-type_domain_containing_protein	7.155	4.812	0.461	0.379
chr6_ + _7967232-7967441	sORF	4.663	6.381	0.406	0.477
chr9_-_4113943-4114041	sORF	16.207	25.106	0.020	0.033

The expression of genes listed in Table 3 is up or down regulated at least two fold in both biological replications.

### Genes upregulated by excess Fe and downregulated by Fe deficiency in shoots

In shoots, 2480 genes were upregulated by excess Fe, of which 1076 (43%) were sORFs (Additional file 2: Table S6). The genes upregulated by excess Fe included a 2-oxoglutarate (OG)-Fe(II) oxygenase domain-containing protein, and an ATPase, and 17 transporter genes belonging to different families. These transporters include two putative plasma membrane ABC transporter domain-containing proteins [a putative subfamily B ABC-type transporter and an MRP-like ABC transporter], two putative amino acid transporters, and two transporters belonging to the multidrug and toxic compound extrusion (MATE) transporter family, which transports small organic compounds (Omote et al. [2006]). The MATE transporter *Os03g0571700* is highly homologous to rice phenolics efflux zero 1, which transports phenolics to solubilize apoplasmic Fe (Ishimaru et al. [2011b]; Ishimaru et al. [2011a]). Additional transporters that putatively transport Cu, magnesium, phosphate or other anions, and oligopeptides were also upregulated (Additional file 2: Table S7). Other genes upregulated by excess Fe include those that participate in cellular metabolic processes, gene expression and translation, and the generation of precursor metabolites and energy (Table 4).

**Table 4:** **Gene ontology analysis of genes upregulated by excess Fe in shoots**

GO ID	GO term	Query	Total	*FDR
GO:0006412	Translation	19	683	2.40E-14
GO:0010467	Gene expression	25	2581	6.40E-09
GO:0044249	Cellular biosynthetic process	29	5899	0.00013
GO:0044267	Cellular protein metabolic process	19	2983	0.00013
GO:0034645	Cellular macromolecule biosynthetic process	24	5248	0.00082
GO:0055086	Nucleobase, nucleoside and nucleotide metabolic	5	275	0.0013
GO:0006091	Generation of precursor metabolites and energy	5	308	0.002
GO:0044237	Cellular metabolic process	32	10813	0.041
GO:0003735	Structural constituent of ribosome	19	455	2.20E-18
GO:0005198	Structural molecule activity	19	531	1.80E-17
GO:0015935	Small ribosomal subunit	17	59	7.60E-31
GO:0030529	Ribonucleoprotein complex	20	503	1.20E-18
GO:0005840	Ribosome	19	456	2.70E-18
GO:0032991	Macromolecular complex	25	1365	3.10E-15
GO:0005737	Cytoplasm	20	1271	1.40E-11
GO:0043228	Non-membrane-bounded organelle	19	1590	2.90E-09
GO:0005622	intracellular	28	4460	5.10E-07
GO:0043226	organelle	22	3164	1.10E-06
GO:0005623	cell	28	6353	0.00015
GO:0043234	protein complex	5	799	0.04

*FDR; False discovery rate.

MapMan analysis revealed that many metabolic related genes were upregulated or downregulated in response to Fe deficiency, and many of these were upregulated in response to excess Fe in shoots (Additional file 1: Figure S3). A total of 43 genes were upregulated by excess Fe and downregulated by Fe deficiency (Figure 2d), of which 9 (21%) were sORFs (Table 5). In shoots, 318 genes were downregulated by Fe deficiency (Additional file 2: Table S7). Interestingly, only one gene (belonging to the cytochrome family) was upregulated in both roots and shoots in response to excess Fe (Figure 2f), whereas 53 genes were downregulated in response to Fe deficiency in both roots and shoots (Figure 2g). Genes that were downregulated by Fe deficiency included Fe sulfur [4Fe-4S] cluster assembly factor, mitochondrial substrate carrier family protein, heavy metal transporters, ferredoxin domain-containing proteins, a bHLH domain-containing protein, heme peroxidases, isocitrate dehydrogenase, *OsNAS3*, a *ferritin* gene, *OsZIP7* and *OsZIP10*, six peroxidases, and 43 sORFs (Additional file 2: Table S7). A summary of the transcriptomic changes in chloroplasts in response to Fe deficiency and excess is shown in Additional file 1: Figure S4. The expression of photosystem II genes was either unchanged or downregulated during Fe deficiency, whereas photosystem I genes were both upregulated and downregulated. In contrast, almost all of the genes involved in ATP synthesis, PS1, and PSII were upregulated in response to excess Fe.

**Table 5:** **Genes upregulated by excess Fe and downregulated by Fe deficiency in shoots**

Locus	Gene	-Fe/+Fe	-Fe/+Fe	++Fe/+Fe	+Fe/+Fe
Os11g0140600	Annexin,_type_VII_family_protein	0.134	0.381	8.732	11.011
LOC_Os03g26100	cDNA transposon protein, putative, unclassified	0.165	0.406	13.344	8.324
LOC_Os05g22840	Conserved_hypothetical_protein	0.154	0.264	4.270	3.790
LOC_Os08g38140	Conserved_hypothetical_protein	0.520	0.542	2.090	2.512
Os01g0559200	Conserved_hypothetical_protein	0.175	0.308	2.070	2.532
Os02g0184100	Conserved_hypothetical_protein	0.447	0.534	2.255	2.249
Os08g0359900	Conserved_hypothetical_protein	0.442	0.448	2.791	3.276
Os05g0556400	DOMON_related_domain_containing_protein	0.415	0.486	2.534	2.959
Os02g0802200	Glycoside_hydrolase_family_79	0.538	0.536	2.711	2.190
Os05g0134400	Heme_peroxidase	0.544	0.530	2.600	2.714
Os02g0135100	NONE";category_"NONE	0.534	0.502	2.722	2.843
Os05g0124900	NONE";category_"NONE	0.041	0.184	4.779	4.932
Os06g0104800	NONE";category_"NONE	0.434	0.449	5.629	8.412
Os07g0407300	NONE";category_"NONE	0.247	0.491	6.269	8.481
Os08g0149701	NONE";category_"NONE	0.208	0.413	2.114	2.395
Os09g0286700	NONE";category_"NONE	0.104	0.388	13.768	14.613
Os09g0332540	NONE";category_"NONE	0.037	0.181	15.617	15.984
LOC_Os09g16320	NONE";category_"NONE	0.037	0.181	15.617	15.984
Os09g0377400	NONE";category_"NONE	0.351	0.390	3.487	4.615
Os10g0330950	NONE";category_"NONE	0.305	0.382	3.141	3.059
Os11g0586700	NONE";category_"NONE	0.121	0.312	2.744	2.357
Os01g0619900	Non-protein_coding_transcript	0.246	0.492	5.356	3.120
Os03g0846250	Non-protein_coding_transcript	0.326	0.541	2.406	2.861
Os01g0720500	OsLhcb1.3	0.298	0.423	2.166	2.575
Os02g0443000	Prefoldin_domain_containing_protein	0.173	0.333	3.485	2.818
Os04g0649900	Protein_of_unknown_function_DUF579,family_protein	0.371	0.546	2.357	2.273
Os01g0909400	Protein_of_unknown_function_DUF868,family_protein	0.349	0.521	2.585	3.464
Os03g0305000	Similar_to_AMP-binding_protein	0.283	0.508	2.729	2.443
Os09g0426800	Similar_to_Glossy1_protein.";category_"II_:	0.260	0.204	2.998	2.006
Os12g0169000	Similar_to_N-acylethanolamine_amidohydrolase	0.434	0.470	2.253	4.054
Os04g0271000	Similar_to_NAD-dependent_deacetylase	0.252	0.505	2.511	2.397
Os04g0538400	Similar_to_Nodulin_21_(N-21)	0.003	0.003	3.385	3.991
Os03g0719900	Similar_to_Peptide_transporter_1	0.455	0.460	4.535	2.624
Os05g0242166	Similar_to_Photosystem_I_reaction_centre_subunit_N	0.210	0.470	2.200	3.465
chr1_-_1443442-1443819	sORF	0.447	0.516	3.455	3.908
chr1_-_10477792-10477944	sORF	0.193	0.308	19.218	21.518
chr3_ + _35148650-35148835	sORF	0.071	0.413	52.628	88.277
chr4_-_7821106-7821402	sORF	0.252	0.464	10.953	12.886
chr4_-_16469013-16469153	sORF	0.142	0.389	2.807	2.190
chr5_ + _8517789-8518034	sORF	0.169	0.077	36.793	13.034
chr7_-_23991237-23991350	sORF	0.171	0.452	35.318	25.773
chr8_ + _9042728-9042955	sORF	0.110	0.366	12.118	9.071
chr9_ + _5568388-5568600	sORF	0.228	0.502	4.912	4.826

The expression of genes listed in Table 5 is up or down regulated at least two fold in both biological replications.

## Discussion

Both Fe deficiency and toxicity cause significant losses in crop yield and quality. In plants, Fe is essential for various cellular processes, as it serves as a cofactor for a range of plant enzymes, including cytochromes, catalase, peroxidase isozymes, ferredoxin, and isozymes of superoxide dismutase (Marschner, [1995]). It was therefore expected that the expression of these genes would be downregulated by Fe deficiency. Genes upregulated during Fe deficiency-associated stress in graminaceous crops have been described extensively (Bashir et al. [2010]; Ishimaru et al. [2009]; Ishimaru et al. [2011b]; Kobayashi et al. [2005]; Nagasaka et al. [2009]; Negishi et al. [2002]; Nozoye et al. [2007]), and our microarray data are consistent with those of previous reports. We have therefore not discussed these genes in detail. Similarly the morphological changes in response to Fe availability as well as the effects of availability of Fe on accumulation of other metals have been widely reported in rice (Ishimaru et al. [2009]; Bashir et al. [2011c]).

Microarray analyses were performed after one week of Fe deficiency and excess treatment and at this point, plants correspond to a new transcriptomic/metabolic steady state. Many genes upregulated by Fe deficiency are also upregulated by other stresses such as cadmium (Egan et al. [2007]) toxicity (Nakanishi et al. [2006]; Takahashi et al. [2011]). Consistent with this, we observed the upregulation of several genes (Additional file 2: Table S1) that are also regulated by other abiotic stresses, including Cd toxicity (Takahashi et al. [2011]) (*OsNRAMP1*), Cu toxicity (Lin et al. [2013]) (*Os04g0588700*, *Os02g0208300*, and *Os04g0512300*), and heat stress (amino acid transporter and heat shock proteins). Many genes reported to be regulated by disease pathogenesis are also upregulated by Fe deficiency (Additional file 2: Table S1). The expression of symbiotic hemoglobin 2 (*rHb2*; *Os03g0226200*), which plays an important role in plant adaptation to unfavorable environment (Zhang et al. [2012a]), was also upregulated by Fe deficiency. These results suggest that Fe-deficient plants undergo oxidative stress, since oxidative stress is common during times of biotic or abiotic stress.

The expression of *1-aminocyclopropane-1-carboxylate oxidase 1* (*Os09g0451400*) was significantly upregulated in Fe-deficient shoots. This gene encodes an intermediate during the formation of ethylene, which plays a role in abiotic stress signaling (Lingam et al. [2011]). Auxins interact with ethylene metabolism, and the expression of four auxin-responsive genes (two auxin-responsive SAUR protein family proteins, one auxin-induced gene, and indoleacetic acid-induced protein 18) was also upregulated by Fe deficiency (Additional file 2: Table S1). These results suggest that ethylene signaling and the reprograming of plant metabolism may be an important strategy of rice in response to Fe deficiency.

### Transcriptomic changes in response to Excess Fe

The expression of several genes was upregulated by excess Fe in roots and shoots. In roots, members of the cytochrome family, oxidases, alcohol dehydrogenase, a protein kinase, a Zn finger domain-containing protein, and a heavy metal transporter were all significantly upregulated. Many of these genes are also regulated by other stresses. For example, the cytochrome_P450_family gene *Os01g0803800* is upregulated by diclofop methyl (Qian et al. [2012]), *Os11g0138300* is regulated by ionizing radiation (Kim et al. [2012]), a heavy metal transporter is regulated by excess silicon and rice blast (Brunings et al. [2009]). One laccase gene that plays a role in lignin formation and two peroxidases (*Os03g0369000* and *Os07g0531400*) are also upregulated by Fe toxicity (Quinet et al. [2012]). These results suggest that under conditions of excess Fe, the generation of reactive oxygen species (ROS) increases, as ROS production is common during times of abiotic or biotic stress. However, it is unknown if the generation of ROS is a direct effect of increased Fe concentrations or is the result of an increased metabolic rate, as suggested by our MapMan analysis.

In shoots, the expression of *OsLhcb1.3* (*Os01g0720500*) was significantly upregulated after treatment with excess Fe. The photosynthetic apparatus of barley adapts to Fe deficiency by remodeling its PSII antenna system, in which the expression of two *Hvlhcb1* genes (*HvLhcb1.11* and *HvLhcb1.12*) is upregulated, and four genes (*HvLhcb1.6-9*) are downregulated by Fe deficiency (Saito et al. [2010]). Although it was not assessed experimentally, it is possible that these downregulated genes would be upregulated in response to excess Fe. Additional genes related to PSII were also upregulated, suggesting that the rate of photosynthesis is increased due to the increased availability of Fe.

The role of ethylene signaling in abiotic stress, including Fe deficiency, has been discussed extensively (Lingam et al. [2011]). Ethylene may also play a significant role in signaling under conditions of excess Fe, since two rice *ethylene response factor-3* (*OsERF3)* genes which regulate ethylene synthesis (Zhang et al. [2013]) were upregulated in shoots in the presence of excess Fe. The expression of *OsRab8A5*, which may be involved in signal transduction, was also upregulated. Upregulation of LONELY GUY, a cytokinin-activating enzyme that regulates activation pathways in rice shoot meristems (Kurakawa et al. [2007]), transcription factors such as *OsMADS18* and *OsMADS56* involved in regulating long-day-dependent flowering (Ryu et al. [2009]) suggest that plant growth and cell division are significantly increased in shoots under conditions of excess Fe. In addition, our MapMan analysis suggested that genes that regulate metabolism are also upregulated in shoots in the response to excess amounts of Fe.

The activity and expression of glutathione reductase (GR) is already reported to change in response to Fe deficiency (Bashir et al. [2007]), while in present experiment upregulation of *OsGR1* was observed in response to excess Fe. Similarly, the expression of NADPH HC toxin reductase, which is reported to be regulated by Cu toxicity (Lin et al. [2013]), also increased by excess Fe. Genes involved in brassinosteroids synthesis were also upregulated. In rice, brassinosteroids regulate multiple developmental processes and modulate several important traits such as height, leaf angle, fertility, and seed filling (Wang et al. [2010]). These results further support the hypothesis that plant metabolism and growth are stimulated under conditions of excess Fe.

The expression of *OsWSL2*, which is associated with the elongation of very long-chain fatty acids, and *Os9BGlu32* was significantly upregulated by excess Fe. Although the function of Os9BGlu32 is unknown, it is a close homolog of Os9BGlu31, which equilibrates the levels of phenolic acids and carboxylated phytohormones and their gluco-conjugates (Luang et al. [2013]). The role of phenolic transport in Fe deficiency has been reported (Bashir et al. [2011b]; Ishimaru et al. [2011b]; Ishimaru et al. [2011a]; Jin et al. [2007]), and it is possible that these phenolics act as antioxidants in the presence of excess Fe. Although the microarray analysis indicates that metabolic rate may increase in response to excess Fe, plants still retain many responses common to different biotic and abiotic stresses. Despite the increased metabolic rate, excess Fe cannot therefore be considered optimal for rice plants, at least under the current growth conditions.

The expression of one 2OG-Fe(II) oxygenase (*Os10g0559500*) was upregulated by Fe deficiency, whereas one gene (*Os08g0392100*) was upregulated by excess Fe. In plants, 2OG-Fe(II) oxygenase are involved in the synthesis of phytosiderophores (Nakanishi et al. [2000]) and numerous other biosynthesis pathways. It was recently suggested that plant 2OG-Fe(II) oxygenases play a role in Fe sensing and metabolism reprograming under Fe-deficient conditions (Vigani et al. [2013]). The upregulation of different 2'-OG dioxygenases by opposing conditions of Fe deficiency and excess suggests that these genes are involved in Fe sensing during altered Fe availability.

### Changes in expression of sORFs in response to Fe deficiency and Excess

In roots, three sORF genes were upregulated by Fe deficiency and downregulated by excess Fe. Our co-expression analysis revealed that these three sORFs are not only positively co-regulated with each other, but also with several other genes presented in Table 1. Specifically, *OsIRO2*, *MIR*, *OPT*, eight conserved hypothetical protein genes, and two *sORF* genes showed a strong positive correlation (*r* < 0.8) when co-expression analysis was carried out for the third sORF (*chr9_-_4113943-4114041*). Seven sORF genes (*chr1_ + _43772594-43772752*, *chr12_-_7456469-7456567*, *chr4_-_24346205-24346330*, *chr4_-_5708578-5708748*, *chr5_ + _27469071-27469241*, *chr6_ + _7967232-7967441*, and *chr9_-_4113943-4114041*) were upregulated by Fe deficiency in both roots and shoots (Additional file 2: Table S8). The upregulation of several sORFs was also confirmed through real time PCR analysis (Figure 3). Among these, very high expression of *chr9_-_4113943-4114041* was observed particularly in shoot tissue (Figure 3b, g). Among these sORFs, the expression of *chr1_ + _43772594-43772752* is not regulated by any other known stresses, according to HanaDB-OS (http://evolver.psc.riken.jp/seiken/OS/index.html), whereas the expression of *chr9_-_4113943-4114041* is significantly downregulated in roots in response to other abiotic stresses such as drought, heat, and salt. It is therefore possible that these sORFs play a significant role (e.g., signalling) during Fe deficiency. Further characterization of these sORFs will help clarify their role in abiotic stress responses.

## Conclusion

Transcriptomic and physiological changes that occur in response to short- and long-term Fe toxicity have been reported (Quinet et al. [2012]). However, our aim was to study the response to excess Fe, and to understand the specific responses of rice to varying Fe concentrations in roots and shoots. Our microarray analysis revealed that cellular metabolism was significantly reprogrammed in response to Fe deficiency and upregulated by excess Fe in shoots even though no morphological changes were observed in shoots under conditions of excess Fe. In addition to the upregulation of genes involved in various metabolic processes, our data suggest increased production of flavonoids and phenols, which may act as antioxidants. The expression of various transporters was also significantly upregulated, which suggests that these transporters coordinate the metabolic changes. Although the responses to Fe deficiency and excess share components with other stress responses, it does not significantly overlap with one particular stress. Moreover, our data reveal that the expression of several sORFs changes with varying Fe availability and that sORFs are co-regulated with other genes involved in Fe deficiency response, suggesting that they are involved in the response to Fe deficiency and/or excess in rice plants. However, the precise function of these sORFs is unclear. Because the products of these sORFs do not contain any characterized domains, it will be challenging to assess their function in response to different abiotic stresses.

It should be noted that the changes in the transcriptome are not specific to Fe, because the concentrations of Cu, Zn, and Mn changed in shoots with perturbations in the Fe level: Cu and Zn were increased during Fe deficiency, while Mn and Cu were increased with excess Fe (Figure 1). As a result, the observed changes in the transcriptome also represent changes in the availability of other metals. Indeed many of the genes reported to be regulated by metal deficiencies such as Zn deficiency changes in response to varying Fe availability (Ishimaru et al. [2012]; Suzuki et al. [2012]; Bashir et al. [2012]; Takahashi et al. [2012]). These analyses also reveal significant information about the regulation of sORFs in response to Fe deficiency and excess. Despite the rapid progress in genomics, uncharacterized and hypothetical genes still represent a large proportion of the rice genome. Understanding the role of these uncharacterized genes, including sORFs, is an important step in comprehensive understanding of the plants' response to different abiotic stresses (Hanada et al. [2007]; Hanada et al. [2010]).

## Methods

### Plant materials and growth conditions

Rice seeds (*Oryza sativa* L. cv. Nipponbare) were germinated for one week at room temperature on paper towels soaked with distilled water. After germination, the seedlings were transferred to a saran net floating on nutrient solution in a glasshouse for one week. Two-week-old plants were transferred to a 20 L plastic box containing nutrient solution with the following composition: 0.7 mM K_2_SO_4_, 0.1 mM KCl, 0.1 mM KH_2_PO_4_, 2.0 mM Ca(NO_3_)_2_, 0.5 mM MgSO_4_, 10 μM H_3_BO_3_, 0.5 μM MnSO_4_, 0.2 μM CuSO_4_, 0.5 μM ZnSO_4_, 0.05 μΜ Na_2_MoO_4_, and 100 μΜ Fe-EDTA as described previously (Suzuki et al. [2006]) and grown for one more week. Plants were grown at 25°C for 14 h of light at 320 μmol photons m^-2^-s^-1^; and at 20°C for 10 h in dark. The nutrient solution was adjusted daily to pH 5.5 with 1 M HCl and was renewed weekly. 30 plants were grown per box (2 plants per hole) and two boxes were prepared for each treatment. For the Fe deficiency and excess treatments, four-week-old plants were transferred to nutrient solution containing 0 (Fe deficiency), 100 (Control), or 500 (excess Fe) μM Fe-EDTA and cultivated for one week. The pH of the nutrient solution was adjusted daily to 5.5, and was renewed weekly. The plants were harvested at noon.

### RT-PCR and microarray analyses

For each treatment, RNA was extracted from six plants in duplicate (two biological replicates, each including six plants). RT-PCR was performed as described previously (Bashir et al. [2011c]), using the primers *OsDMAS1* RT (forward) 5`-GCCGGCATCCCGCAGCGGAAGATCA-3' and *OsDMAS1* RT (reverse) 5`-CTCTCTCTCTCGCACGTGCTAGCGT-3'. The primers used to assess *osvit2* by RT-PCR (qRT-PCR) were (forward) 5`-AAGGCCTGGCTCGAATTCATG-3' and (reverse) 5`-GTGTATTAGATGTTCTGGAGGTG-3'. The *α-tubulin* primers used were (forward) 5`-TCTTCCACCCTGAGCAGCTC-3' and (reverse) 5`-AACCTTGGAGACCAGTGCAG-3'. Primers used for real time PCR were as follows *OsDMAS1*, (forward) 5`-GAGGAGGAGAGGCAGAGGAT-3' and (reverse) 5`-TCAACACGATCGTCAAGAGC-3', *OsFRO2* (forward) 5`-GCCAGATGTTCGAGCTCTTC-3' and (reverse) 5`-GGGCTTTTGCAGAAGTTGAG-3', *Os01g0127000* (forward) 5`-GAGAACATGACGAGCAACGA-3' and (reverse) 5`-AGCATGCAGCTCTTGAAGGT-3', *Os07g0142100* (forward) 5`-CGTCTTCCTCGATAGCCAAA-3' and (reverse) 5`-AGCTGGAGCCACATCGAC-3', *chr6_ + _23392831-23392944* (forward) 5`-TCGTGTGTAATAATATGGGCTGTT-3' and (reverse) 5`-GGATACAATGGGAAATGAGCA-3', *chr6_ + _29900249-29900395* (forward) 5`-CACACGTGCGAGATCTACCT-3' and (reverse) 5`-AAAGGAAAGATTGCCATCCA-3', *chr7_-_23991237-23991350* (forward) 5`-ATGTTCTACCCCATGCCACT-3' and (reverse) 5`-ATGTCGCTGGACACCCTAAC-3', *chr9_-_4113943-4114041* were (forward) 5`-GGCCTGTGCTAGTTTTGGTG-3' and (reverse) 5`-ATGGGCGCAAATTACATCAT-3' respectively. All experiments were performed in a minimum of triplicates.

The microarray slides were custom-designed and contained 101,720, 60 mer probes. Of these, 48,620 were for sORFs, 50,962 probes represented RAP-DB, and the rest belonged to TIGR. For our microarray analysis, RNA was labelled using an Agilent Low RNA Input Linear Amplification Kit (Agilent Technologies Inc., Santa Clara, CA), following the manufacturer's instructions. The microarray analyses were performed as described previously (Hanada et al. [2013]) with the exception that two biological replicates were used. Data analysis was performed using Feature Extraction and Image Analysis software (Agilent Technologies Inc.) and Microarray Suite (Affymetrix, Santa Clara, CA), and normalized and processed as described (Hanada et al. [2013]). Those genes with a low signal intensity (<300) were filtered to focus on genes that were highly expressed under conditions of Fe deficiency and excess. For our MapMan analysis, the average log_2_ value of both biological replicates was calculated for individual annotations in response to Fe deficiency and excess in roots and shoots. This log_2_ value was then used to compare the transcriptomic changes in metabolism-related genes using MapMan 3.5.1R2 (Thimm et al. [2004]). Our gene ontology analyses were carried out at http://www.geneontology.org/. Coexpression analyses were done at http://evolver.psc.riken.jp/seiken/OS/co-express.html. This database contains microarray data of 40 different experimental conditions obtained through microarray analysis using the same custom microarray chip as described in this manuscript.

### Determination of metal concentrations

Roots were washed with de-ionized water before harvesting. Leaf and root samples were dried for three days at 70°C, and then digested with 3 ml of 13 M HNO_3_ at 220 C for 40 min using a MARS XPRESS microwave reaction system (CEM, Matthews, NC). All samples were processed with four biological replicates. After digestion, the samples were collected, diluted to 5 ml, and analyzed by ICP-AES (SPS1200VR; Seiko, Tokyo, Japan), as described previously (Ishimaru et al. [2011b]; Ishimaru et al. [2007]).

### Recording of the morphological characteristics of the plants

Root and shoot lengths were measured using a scale. The degree of chlorosis in the youngest fully expanded leaf was determined using a SPAD-502 chlorophyll meter (Minolta Co., Tokyo, Japan), as described previously (Ishimaru et al. [2012]).

## Authors' contributions

KB, KH and NN designed the study, KB, MS and KH performed the research, and KB, KH, MS, HN and NN discussed the data and wrote the manuscript. All authors read and approved the final manuscript.

## Additional files

## Electronic supplementary material

Additional file 1: Figure S1.: Expression of *OsVIT2* and *OsDMAS1* under Fe deficiency and excess Fe. **Figure S2.** Transcriptional changes in metabolism related genes in roots of Fe-deficient and excess Fe rice as predicted by MapMan 3.5.1R2. **Figure S3.** Transcriptional changes in metabolism related genes in shoots of Fe-deficient and excess Fe rice as predicted by MapMan 3.5.1R2. **Figure S4.** Summary of transcriptional changes in chloroplast of Fe-deficient and excess Fe rice shoots as predicted by MapMan 3.5.1R2. (PDF 2 MB)

Additional file 2: Table S1.: Genes upgegulated by Fe deficiency in roots. **Table S2.** Genes down-gegulated by Fe excess in roots. **Table S3.** Genes down-regulated by Fe Deficiency. **Table S4.** Genes upregulated by Fe deficiency in shoots. **Table S5.** Genes down regulated by Fe excess in shoots. **Table S6.** Genes upregulated by Fe excess in shoots. **Table S7.** Genes down-regulated by Fe deficiency in shoots. **Table S8.** Genes upregulated by Fe deficiency in roots and shoots. (XLS 2 MB)

Below are the links to the authors’ original submitted files for images.

Authors’ original file for figure 1

Authors’ original file for figure 2

Authors’ original file for figure 3

## Abbreviations

Fe: Iron
sORF: Small open reading frames
