# Supplementary material for: Transcriptomic analysis of rice in response to iron deficiency and excess
Source: Rice (N Y). 2014 Sep 12;7:18. doi: 10.1186/s12284-014-0018-1 (PMC4884027; doi:10.1186/s12284-014-0018-1)
Supplement: Supplementary file 1 — Additional file 1: Figure S1.: Expression of OsVIT2 and OsDMAS1 under Fe deficiency and excess Fe. Figure S2. Transcriptional changes in metabolism related genes in roots of Fe-deficient and excess Fe rice as predicted by MapMan 3.5.1R2. Figure S3. Transcriptional changes in metabolism related genes in shoots of Fe-deficient and excess Fe rice as predicted by MapMan 3.5.1R2. Figure S4. Summary of transcriptional changes in chloroplast of Fe-deficient and excess Fe rice shoots as predicted by MapMan 3.5.1R2. (PDF 2 MB) [file 12284_2014_18_MOESM1_ESM.pdf]

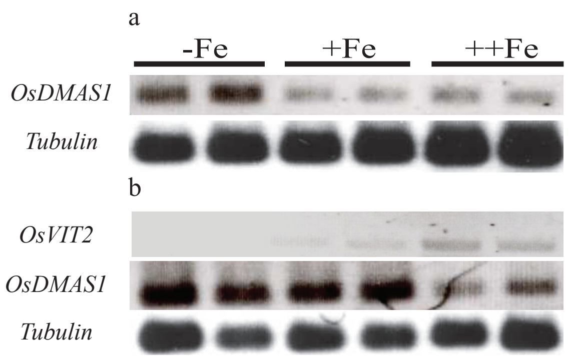


**Supplementary Fig.-1. Expression of *OsVIT2* and *OsDMAS1* under Fe deficiency and excess Fe**

a). Expression of *OsDMAS1* and *tubulin* in roots

b). Expression of *OsVIT2*, *OsDMAS1* and *tubulin* in shoots

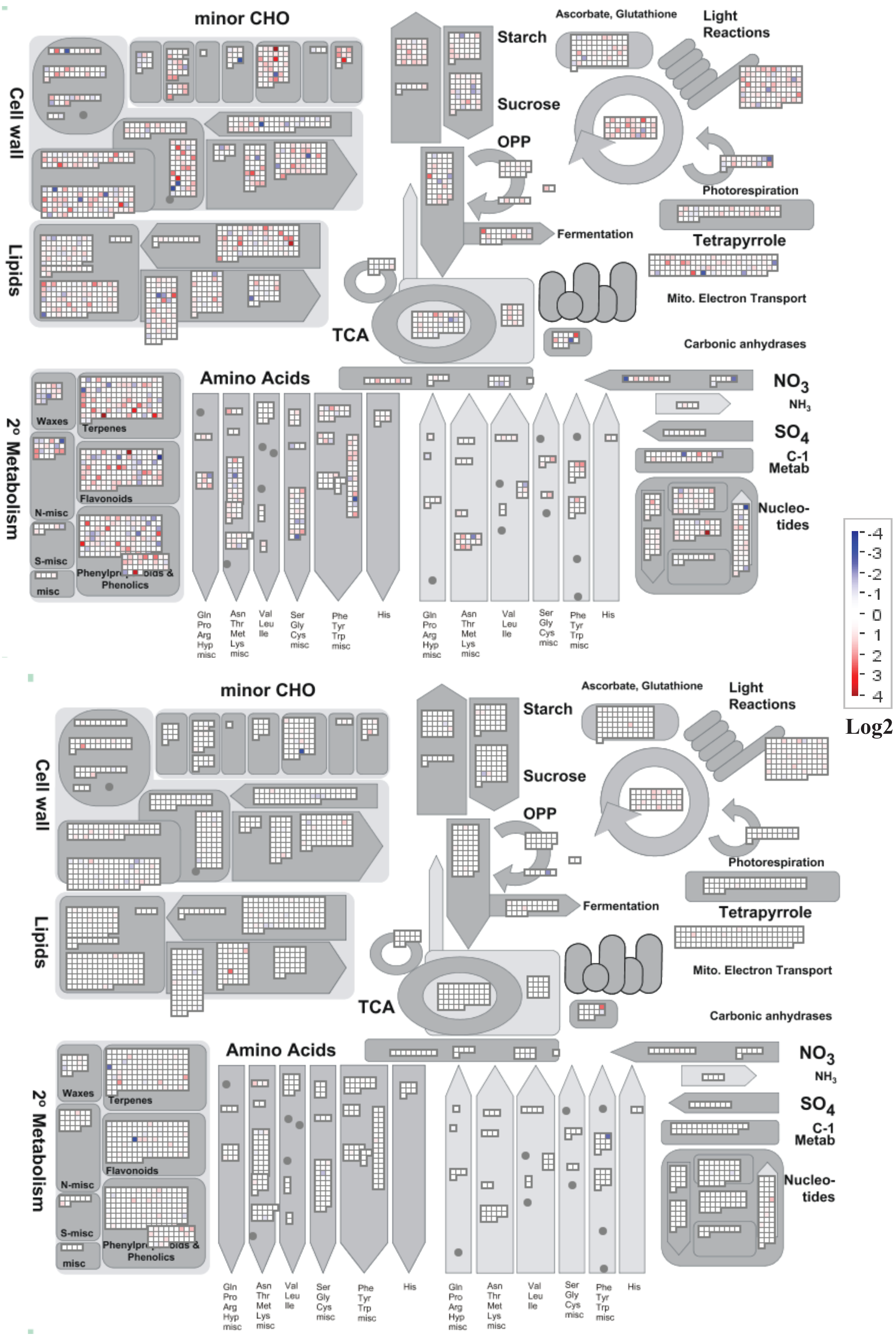


**Supplementary Fig.-2. Transcriptional changes in metabolism related genes in roots of Fe-deficient and excess Fe rice as predicted by MapMan 3.5.1R2.**

a). Transcriptional changes in response to Fe-deficiency.
b). Transcriptional changes in response to excess Fe.
Dots shown in red represent upregulated genes, while dots shown in blue represent downregulated genes.

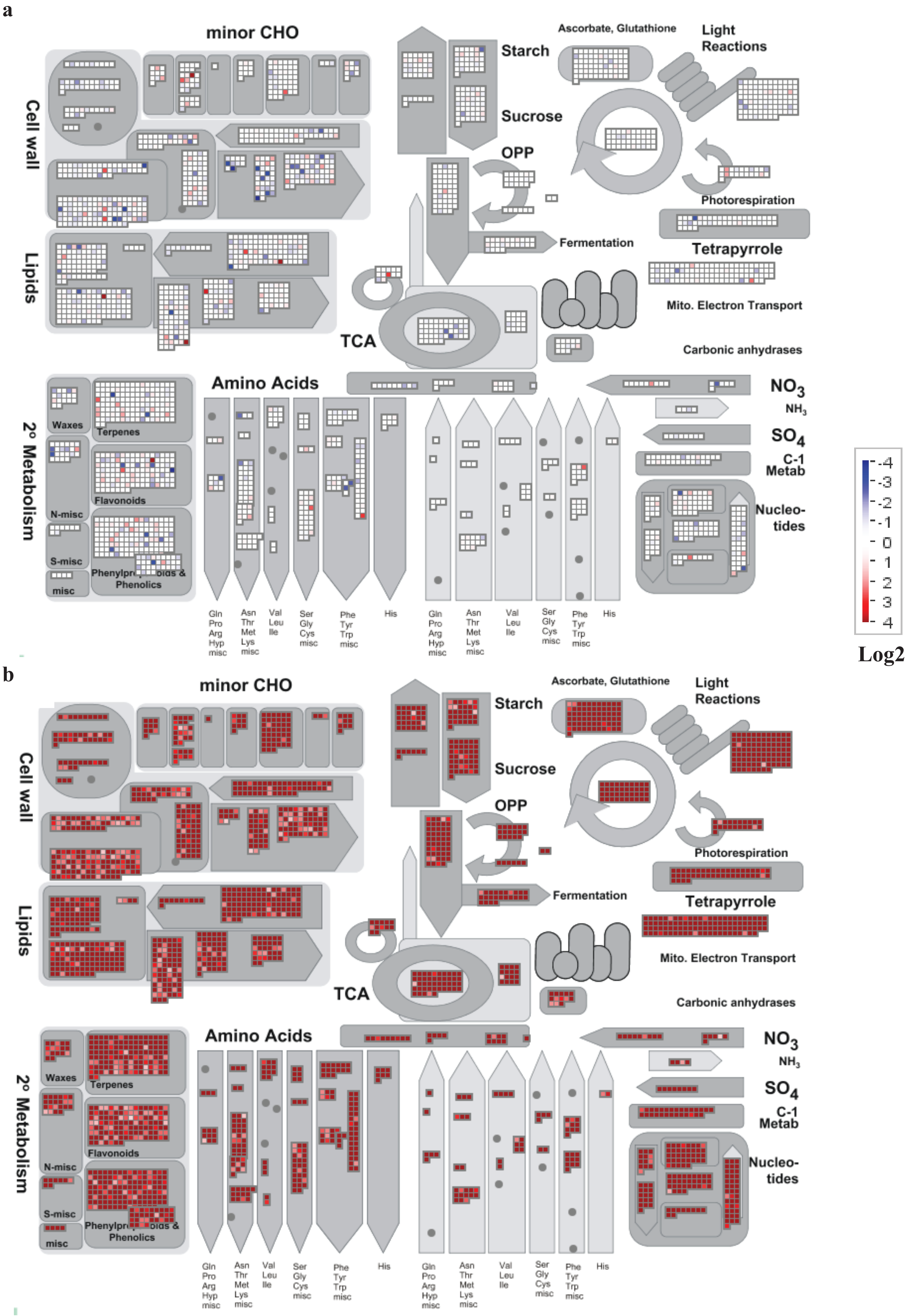


**Supplementary Fig.-3. Transcriptional changes in metabolism related genes in shoots of Fe-deficient and excess Fe rice as predicted by MapMan 3.5.1R2.**

a). Transcriptional changes in response to Fe-deficiency.

b). Transcriptional changes in response to excess Fe.

Dots shown in red represent upregulated genes, while dots shown in blue represent downregulated genes.

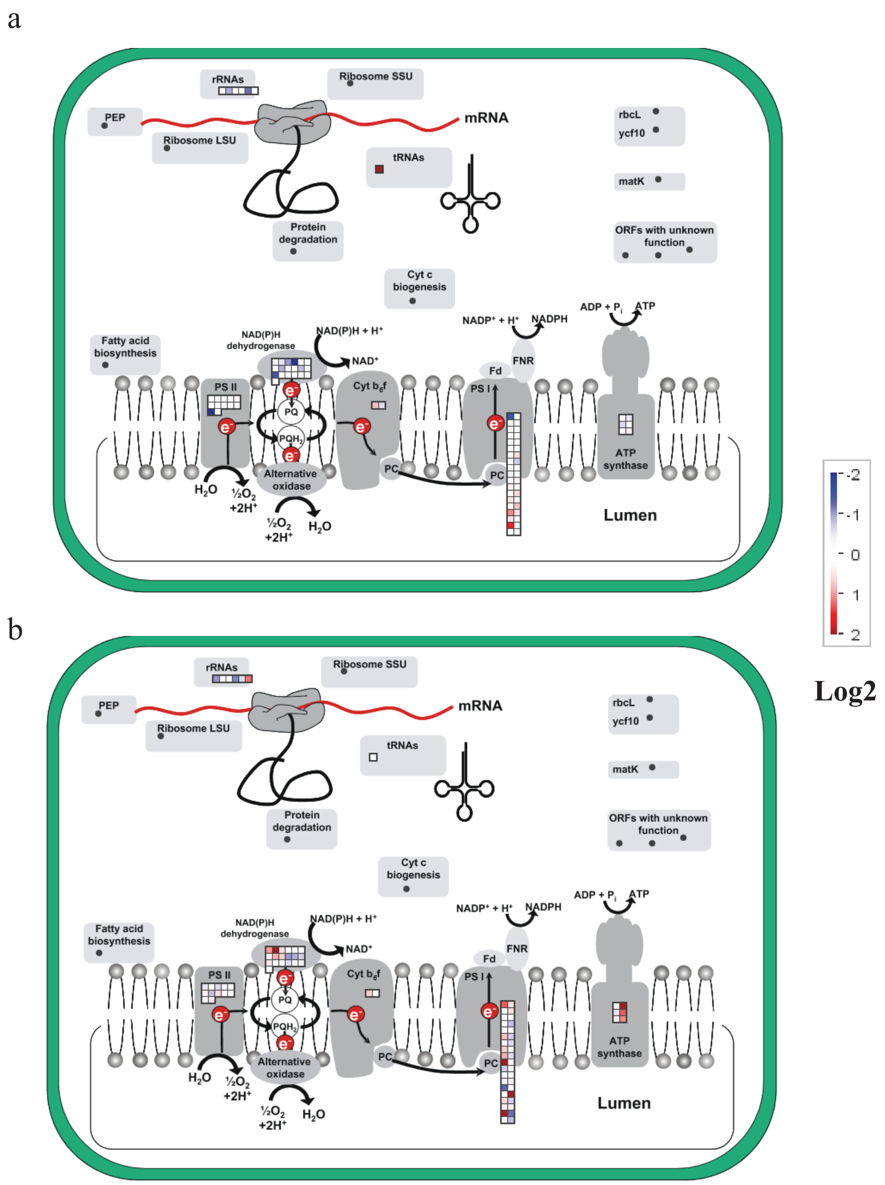


**Supplementary Fig.-4. Summary of transcriptional changes in chloroplast of Fe-deficient and excess Fe rice shoots as predicted by MapMan 3.5.1R2.**

a). Transcriptional changes in response to Fe-deficiency.

b). Transcriptional changes in response to excess Fe.

Dots shown in red represent upregulated genes, while dots shown in blue represent downregulated genes.
