# Supplementary figures and images for: Transcriptomic analysis of rice in response to iron deficiency and excess
Source: Rice (N Y). 2014 Sep 12;7:18. doi: 10.1186/s12284-014-0018-1 (PMC4884027; doi:10.1186/s12284-014-0018-1)

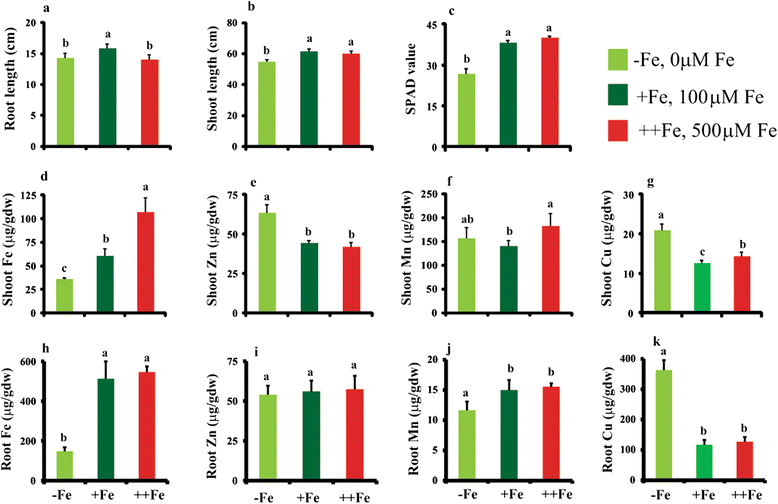

Supplement: Supplementary file 3 — Authors’ original file for figure 1 [file 12284_2014_18_MOESM3_ESM.gif]

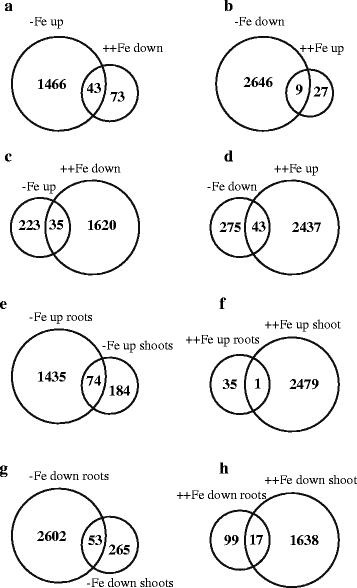

Supplement: Supplementary file 4 — Authors’ original file for figure 2 [file 12284_2014_18_MOESM4_ESM.gif]

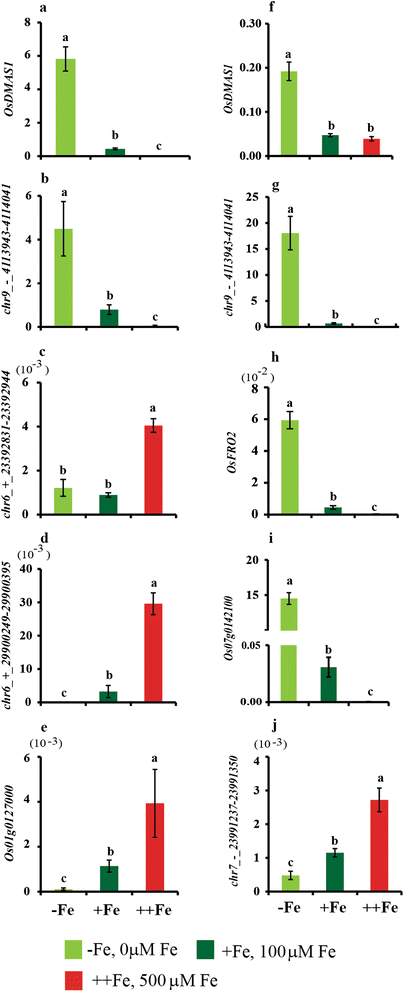

Supplement: Supplementary file 5 — Authors’ original file for figure 3 [file 12284_2014_18_MOESM5_ESM.gif]
